# The role of gut microbiome disruption in the development of metabolic dysfunction-associated kidney disease

**DOI:** 10.3389/abp.2026.16436

**Published:** 2026-07-24

**Authors:** Michał Witkowski, Jarosław Przybyciński, Bartosz Wojciuk, Wiktor Czaja, Natalia Gołembiewska, Edyta Gołembiewska

**Affiliations:** 1 Department of Nephrology, Transplantology and Internal Medicine, Pomeranian Medical University, Szczecin, Poland; 2 Department of Microbiology, Immunology and Laboratory Medicine, Pomeranian Medical University, Szczecin, Poland; 3 Uniwersytet Medyczny im Karola Marcinkowskiego w Poznaniu, Wydział Medyczny, Poznań, Poland

**Keywords:** cardiovascular disease, gut microbiota, MDAKD, obesity, type 2 diabetes

## Abstract

Metabolic dysfunction-associated kidney disease (MDAKD) is increasingly recognised as a major clinical consequence of the global rise in obesity, type 2 diabetes, hypertension, and cardiovascular disease. Accumulating evidence suggests that the gut microbiota may contribute to the development and progression of metabolic and renal disorders through complex mechanisms involving microbial metabolites, immune activation, and disruption of the intestinal barrier. This review provides an overview of current knowledge regarding the role of the gut microbiota and gut-derived metabolites in the pathogenesis of chronic kidney disease (CKD) associated with metabolic disorders. Special attention is given to short-chain fatty acids, bile acids, N-trimethylamine oxide, branched-chain amino acids, indoxyl sulfate, p-cresol sulfate, and lipopolysaccharides. Accumulating experimental and clinical evidence suggests that dysbiosis may contribute to chronic low-grade inflammation, insulin resistance, endothelial dysfunction, lipotoxicity, and profibrotic signaling pathways associated with kidney injury and cardiovascular complications. The review also identifies significant limitations in current microbiome research, such as the predominance of animal studies, methodological challenges in metabolite quantification, and difficulties in establishing causality in humans. Emerging therapeutic strategies targeting the gut microbiota, including dietary interventions, prebiotics, probiotics, sodium-glucose cotransporter 2 inhibitors, glucagon-like peptide-1 receptor agonists, and faecal microbiota transplantation, may offer novel approaches to slowing CKD progression and improving metabolic health. However, further mechanistic and clinical studies are required to determine the efficacy of microbiota-targeted therapies in MDAKD.

## Introduction

The gut microbiota constitutes a complex ecosystem within the human gastrointestinal tract, containing approximately 10^11^ bacteria per Gram in the colon and composed primarily of members of the phyla *Bacteroidota* (formerly *Bacteroidetes*) and *Bacillota* (formerly *Firmicutes*) (Sender et al., 2016). This microbial community plays a critical role in host nutrition, metabolism and immune responses, and facilitates continuous, bidirectional communication between multiple organs. Despite recent advances, the gut-kidney axis remains insufficiently explored (Tsuji et al., 2024). The clinical importance of this axis is highlighted by the global prevalence of cardiovascular diseases (CVD), which frequently co-occur with chronic kidney disease and metabolic disorders such as type 2 diabetes (T2D) and obesity. In 2024, an estimated 589 million adults worldwide were affected by diabetes, resulting in approximately 3.4 million deaths, underscoring the substantial public health burden of these conditions (IDF, 2025). Additionally, the cluster of risk factors defining metabolic syndrome—abdominal obesity, hypertension, and hyperglycemia—is associated with a marked increase in the risk of developing both T2D and CVD (Lin et al., 2022). The convergence of these systemic metabolic disorders has led to the identification of metabolic dysfunction-associated kidney disease (MDAKD), a distinct clinical entity in which kidney dysfunction arises from the combined effects of insulin resistance, lipotoxicity and chronic systemic inflammation (Bansal and Chonchol, 2025). Substantial evidence indicates that alterations in the gut microbiota, termed dysbiosis, may contribute to the pathogenesis of these chronic conditions (Liu et al., 2025). Dysbiosis may serve as a significant co-factor in the development of MDAKD and, in cases of clinically significant T2D, may also accelerate progression to diabetic kidney disease (DKD), which is recognized as the leading cause of end-stage renal disease (ESRD) globally. This microbial imbalance initiates several pathological pathways, including metabolic dysregulation, immune activation, and impairment of the intestinal barrier (Fei et al., 2025). These disturbances can mediate kidney injury by systemic translocation of harmful microbial metabolites and by maintaining a persistent inflammatory state, thereby promoting the onset and progression of MDAKD (Jiang H. et al., 2025). Elucidating the specific pathogenic mechanisms by which the gut microbiota and its metabolites affect renal function is therefore essential for developing novel and effective therapies to slow the progression of this metabolic-renal syndrome (Mao et al., 2023).

This review aims to outline the interactions among gut microbiota, metabolic disorders, and the development and progression of CKD, with particular emphasis on the role of microbiota-derived metabolites.

## Development of chronic kidney disease in metabolic disorders

There is growing interest in understanding the role of metabolic disorders in the development of chronic kidney disease, now recognized as a component of the newly defined clinical syndrome known as MDAKD. MDAKD refers to chronic kidney disease in individuals with metabolic disorders such as diabetes, insulin resistance, obesity, or hypertension (Bansal and Chonchol, 2025) and emphasizes the causal relationship between metabolic disorders and kidney disease. The mechanisms underlying metabolic disorder-induced kidney damage are complex and remain incompletely understood. This chapter reviews the principal pathways linking metabolic disorders to kidney damage.

### Obesity and CKD

Obesity is a recognized and independent risk factor for chronic kidney disease. Accumulation of visceral fat within the abdominal cavity is a defining characteristic of metabolic syndrome and is associated with an increased risk of kidney disease (Stasi et al., 2022). This process involves multiple mechanisms, including hemodynamic alterations, lipid metabolism disturbances, and hormonal dysregulation. Elevated visceral fat, resulting from increased leptin production, stimulates adipocytes to overproduce aldosterone and modifies mineralocorticoid receptor activation (Vecchiola et al., 2016). Activation of the renin-angiotensin-aldosterone system (RAAS) and heightened sympathetic activity in individuals with obesity further elevates aldosterone levels (Sharma and Engeli, 2006). Aldosterone enhances sodium reabsorption in renal tubules, leading to sodium and water retention. Additionally, RAAS activation contributes to glomerular hyperfiltration by exerting a greater effect on efferent arterioles compared to afferent arterioles (Navar, 2014). To compensate for the increased glomerular filtration rate (GFR) observed in obesity, glomerular hypertrophy (glomerulomegaly) develops, followed by mechanical stretching of podocytes, which are unable to accommodate the increased glomerular volume (Chagnac et al., 2019). When podocytes reach their stretching limit, their foot processes detach from the glomerular basement membrane. This event leads to the development of focal sclerotic changes, termed obesity-associated glomerulopathy in the context of obesity (Zhang and Lerman, 2017). At this stage, obesity-related kidney damage becomes clinically evident, presenting as proteinuria and/or reduced GFR in laboratory assessments (Salvatore et al., 2017). Weight gain also increases perirenal adipose tissue, which may compress the thin segment of the loop of Henle and the vasa recta vessels in the renal medulla, thereby reducing renal tubular flow. Reduced tubular flow, combined with RAAS activation, decreases sodium excretion and leads to secondary glomerular hyperfiltration (Parvanova et al., 2023). Compression of the kidney further stimulates juxtaglomerular apparatus cells in the macula densa to release renin, amplifying RAAS activation. Concurrently, increased perirenal adipose tissue increases intrarenal pressure, leading to oedema and renal tissue inflammation (Kanbay et al., 2025). In obesity, fat also accumulates in the renal interstitium, a condition known as fatty kidney, which contributes to compression of renal vessels, increased intrarenal pressure, and further RAAS activation, ultimately causing kidney damage (Parvanova et al., 2023). Adipokines released by renal fat cells promote renal parenchymal inflammation and fibrosis, primarily by upregulating transforming growth factor beta (TGF-β1) expression (Abdullah et al., 2025). Increased fatty acid availability also leads to greater uptake by podocytes and tubular cells, potentially inducing apoptosis when the cellular capacity for lipid oxidation or storage is exceeded (Schelling, 2022).

### Insulin resistance, diabetes mellitus, and CKD

Diabetes represents the primary cause of chronic kidney disease in numerous countries. Kidney damage may occur as early as the development of insulin resistance, preceding the onset of overt diabetic symptoms. Similar to obesity, insulin resistance induces sodium retention and vascular endothelial contraction, which in turn activates the RAAS (Sarafidis and Ruilope, 2006). Insulin resistance also promotes lipid droplet accumulation in renal tubular cells by increasing the activity of sterol regulatory element-binding protein 1c (SREBP-1c), ultimately leading to tubular atrophy and renal interstitial fibrosis (Li et al., 2024). In the initial stages DKD, glomerular hyperfiltration develops due to impaired regulation of afferent and efferent arteriolar tone, a consequence of increased glucose uptake in proximal tubular cells (Vallon and Thomson, 2020). Prolonged dilatation of the afferent arteriole in the context of elevated glucose uptake may lead to glomerular enlargement and proliferation of mesangial cells and the glomerular basement membrane (Chagnac et al., 2019). Hyperglycemia is a critical factor in the pathogenesis of diabetic kidney damage. Hyperglycemia increases endothelin-1 production, a potent vasoconstrictor (Park et al., 2000). Elevated blood glucose and endothelin-1 levels contribute to podocyte cytoskeletal breakdown and subsequent apoptosis (Hu et al., 2024). In hyperglycemic states, proximal tubular cells increase glucose uptake, a process that is energetically demanding. These cells rely exclusively on fatty acids as an energy source. Increased ATP consumption for glucose transport leads to hypoxia in proximal tubular cells, activating the hypoxia-inducible factor (HIF) and AMP-activated protein kinase (AMPK) pathways and resulting in hypertrophy of these cells (Mohandes et al., 2023). Persistent hyperglycemia induces a metabolic shift to the glycolytic pathway in these cells (Zhang et al., 2018). Inefficient fatty acid oxidation in the proximal tubules, due to abnormal energy expenditure and metabolic alterations, is considered the primary cause of renal tubular damage and fibrosis in diabetes (Lv et al., 2025).

### Hypertension and CKD

Elevated blood pressure, similar to the aforementioned metabolic changes, contributes to kidney damage and the progression of chronic kidney disease (Lin et al., 2024). Furthermore, metabolic disorders such as obesity and diabetes promote the onset of hypertension, primarily through excessive sympathetic nervous system activity and activation of the RAAS (Kalil and Haynes, 2012). Glomerular hyperfiltration is the principal factor accelerating kidney damage in hypertension. Mechanisms underlying this process include reduced nephron number, increased sodium retention and subsequent extracellular volume expansion, vascular endothelial dysfunction, and activation of both the RAAS and the sympathetic nervous system (Bansal and Chonchol, 2025). As hypertension advances, renal blood flow diminishes, predominantly affecting the renal tubules. Resulting ischemia further enhances angiotensin II synthesis, which exerts vasoconstrictive effects and stimulates renal parenchymal cell proliferation, thereby worsening both hemodynamic and extravascular injury, including fibrosis (Basile et al., 2012).

Subsequent sections examine the associations between gut microbiota and obesity, T2D, and CVD, with particular emphasis on the mediating role of microbiota-derived metabolites in the potential development of these conditions.

## Gut microbiota and obesity

Overweight and obesity can be associated with disruption in gut microbiota diversity and function, referred to as dysbiosis. Multiple studies, first in animal models and subsequently in obese individuals, have identified alterations in the relative abundance of two bacterial phyla: *Bacillota* and *Bacteroidota.* Most studies report an increased proportion of *Bacillota* and a concurrent decrease in *Bacteroidota* (Kallus and Brandt, 2012). Additionally, individuals with obesity often display reduced gut microbiota diversity (Díaz Perdigones et al., 2025). Despite pending research, a definitive microbial pattern of obesity has not yet been determined. Current evidence suggests the gut microbiota may influence obesity development (Sasidharan Pillai et al., 2024). Possible mechanisms associated with gut dysbiosis that may promote obesity include imbalances in energy homeostasis, increased fat synthesis and storage, and altered appetite regulation. However, these findings remain inconclusive and have not been confirmed by later studies (Magne et al., 2020). Additionally, randomized human trials of gut microbiota transplantation have shown no effect on body weight (Dalby, 2021). The following section outlines proposed mechanisms linking altered gut microbiota to obesity. Importantly, most mechanistic evidence originates from animal studies, whereas evidence supporting these pathways in humans remains limited. Therefore, the mechanisms described below should be interpreted as biologically plausible hypotheses rather than established causal pathways in human obesity.

### Changes in energy balance

Studies in mice have shown that obesity-associated alterations in gut microbiota composition may increase energy harvest from ingested food (Turnbaugh et al., 2006). Proposed mechanisms include expansion of specific bacterial taxa, such as *Thomasclavelia ramosa* (formerly *Clostridium ramosum*), which has been associated with increased intestinal expression of nutrient transporters including glucose transporter 2 (GLUT2) and CD36 (a fatty acid translocase) (Woting et al., 2014). Obesity-related microbiota has also been linked to an increased abundance of microbial enzymes involved in polysaccharide degradation, potentially enhancing fermentation and production of short-chain fatty acids (SCFAs) (Turnbaugh et al., 2006). In addition, interactions between hydrogen-producing bacteria and methanogenic archaea may increase fermentation efficiency and SCFA generation (Zhang et al., 2009). In experimental animal models, increased energy harvest is not necessarily accompanied by a proportional increase in energy expenditure. Proposed explanations include alterations in bile acid metabolism and SCFA signaling. In obese mice, changes in gut microbiota composition have been associated with reduced production of secondary bile acids and altered activation of TGR5- and FXR-dependent pathways, potentially leading to impaired thermogenesis and reduced browning of white adipose tissue (Gao et al., 2025). Early studies in mice suggested that the gut microbiota may promote energy storage by suppressing intestinal fasting-induced adipose factor (FIAF/ANGPTL4) expression, thereby facilitating lipid accumulation (Bäckhed et al., 2004). However, subsequent studies in human intestinal cell models demonstrated that SCFAs can induce ANGPTL4/FIAF expression via PPARγ activation (Alex et al., 2013), suggesting that the relationship among SCFAs, FIAF and energy metabolism is more complex than initially proposed. In addition, butyrate has been shown to increase PGC-1α and UCP1 expression and enhance thermogenesis in mice (Gao et al., 2009). Overall, the effects of SCFAs on energy expenditure remain incompletely understood. Taken together, evidence from rodent studies suggests that obesity-associated alterations in gut microbial composition may influence energy balance through effects on nutrient harvest, bile acid metabolism and SCFA signaling. However, these mechanisms have been demonstrated predominantly in animal models, and direct evidence that they make a substantial contribution to obesity development in humans remains limited.

### Promotion of fat production and storage

Alterations in bile acid metabolism have been associated with obesity and obesity-related changes in gut microbiota composition. In murine models, obesity-associated dysbiosis has been linked to changed microbial conversion of primary to secondary bile acids, resulting in shifts in bile acid composition (Sayin et al., 2013). These compositional changes can influence bile acid–receptor signaling, including FXR pathways, since FXR activity is differentially regulated by specific bile acid species, thereby affecting hepatic lipid and glucose metabolism, as demonstrated in mouse studies (Trauner et al., 2010). In experimental mouse models, alterations in FXR signaling have been associated with changes in intestinal FGF15/19 production and downstream effects on hepatic lipid metabolism. Disruption of this signaling axis may contribute to increased lipogenic pathways, although the underlying mechanisms are complex and involve multiple regulatory steps (Bhatnagar et al., 2009). The relevance of these pathways to human obesity remains uncertain. SCFAs can contribute to gluconeogenesis and serve as substrates for lipid synthesis, acting as precursors for the synthesis of fatty acids and cholesterol (Amabebe et al., 2020). In contrast, studies in mice have identified an association between butyric acid and reduced lipogenesis, mediated by inhibition of peroxisome proliferator-activated receptor γ (PPAR-γ) activity and stimulation of β-oxidation (Zheng et al., 2023). Collectively, findings from experimental animal studies underscore the complexity of SCFA-mediated regulation of lipid metabolism. Although these pathways may influence fat accumulation under experimental conditions, their quantitative contribution to obesity development in humans remains unclear.

### Regulation of the appetite

Alterations in the gut microbiota can also affect appetite regulation. Evidence from rat models indicates that the production of gut hormones, including peptide YY (PYY) and glucagon-like peptide 1 (GLP-1), is closely associated with the presence of bile acids and SCFAs (Sun et al., 2018; Christiansen et al., 2018). Mouse studies demonstrate that increased SCFA production promotes satiety and reduces appetite, potentially through activation of G protein-coupled receptors 41 (GPR41) and 43 (GPR43). In mouse studies, activation of these receptors enhances the production and secretion of appetite-suppressing gut hormones, specifically GLP-1 and PYY (Cunningham et al., 2021). Notably, concentrations of these hormones are often reduced or dysregulated in obese individuals. SCFAs may also exert central effects by acting directly in the hypothalamus. In mouse models, SCFAs induce the expression of α-melanocyte-stimulating hormone, which has an anorexigenic effect, while simultaneously inhibiting the expression of agouti-related protein (AGRP), which has an orexigenic effect (Frost et al., 2014). The gut microbiota may modulate host neurochemical pathways implicated in appetite regulation, such as γ-aminobutyric acid (GABA) signaling and serotonin metabolism. Specific intestinal microorganisms can produce GABA, and the gut microbiota can influence host serotonin synthesis and tryptophan metabolism (Yano et al., 2015; Strandwitz et al., 2019). Nevertheless, neither peripheral serotonin nor microbiota-derived serotonin crosses the blood-brain barrier to a significant degree under normal physiological conditions (Berger et al., 2009). Likewise, direct transport of microbiota-derived GABA into the central nervous system has not been established. Consequently, the influence of the gut microbiota on central appetite regulation is thought to act indirectly, for example, by modulating tryptophan availability, vagal signaling, immune pathways, or gut hormone secretion (Morais et al., 2021; Cryan et al., 2019). Most evidence supporting these mechanisms derives from animal studies, and direct evidence that microbiota-induced neurochemical changes substantially influence appetite regulation in humans remains limited.

### Challenges and limitations in studying gut microbiota among individuals with obesity

Although numerous studies have reported associations between obesity and gut microbiota composition, caution is warranted regarding the immediate practical application of these findings. This is particularly important because much of the mechanistic evidence discussed in the literature originates from experimental animal models rather than human intervention studies. This caution is necessary due to persistent challenges in interpreting microbiota research. For example, identification of microbiota components is typically conducted at various taxonomic levels, often resulting in conclusions based on higher taxa rather than specific species. The widely reported imbalance in the *Bacteroidetes/Firmicutes* (now *Bacteroidota/Bacillota*) ratio involves two broad bacterial groups, each encompassing multiple species. Additionally, studies investigating specific bacterial species as potential therapeutic agents frequently lack mechanistic explanations. Moreover, even rigorous animal experiments may not adequately distinguish the bidirectional interactions between diet and microbiota (Cani et al., 2021; Arrieta et al., 2016).

The mechanisms outlined above have been demonstrated predominantly in rodent models and should therefore be interpreted with caution. Although associations between obesity and gut microbiota composition have been reported in humans, causal pathways remain poorly established. Furthermore, randomized clinical studies, including fecal microbiota transplantation trials, have generally failed to demonstrate meaningful effects on body weight. Consequently, the translational relevance of many experimentally described mechanisms remains uncertain and requires confirmation in well-controlled human studies.

## Gut microbiota and T2D

Alterations in gut microbiota composition have been demonstrated in both animal models and humans with T2D. These alterations primarily affect microbial diversity at the phylum and class levels. However, no consistent microbiota profile has yet been identified as associated with the development of T2D. Similar to observations in obesity, individuals with diabetes typically exhibit increased abundance of the Bacillota and Pseudomonadota phyla, along with a concurrent decrease in Bacteroidota abundance (Li et al., 2020). Opportunistic microorganisms, including *Bacteroides caccae*, *Hungatella hathewayi*, *Thomasclavelia ramosa*, *Clostridium symbiosum*, *Eggerthella lenta,* and *Escherichia coli,* are frequently detected in the gut microbiome of individuals with T2D. In a comparative study of healthy individuals and those diagnosed with T2D, *Akkermansia muciniphila* and *Faecalibacterium prausnitzii* were found to confer protection against the development of T2D (Park et al., 2023). *Akkermansia* species play a central role in maintaining the integrity of the mucin layer and reducing inflammation, and reduced numbers of *Akkermansia* colonies have been observed in patients with diabetes (Zhang et al., 2021). *Faecalibacterium prausnitzii* appears to reduce systemic inflammation and improve insulin sensitivity, as demonstrated in mouse models (Xuan et al., 2023). The following section outlines potential mechanisms by which gut microbiota metabolites may contribute to the development of insulin resistance and, consequently, type 2 diabetes.

### SCFAs

Patients with T2D often exhibit a reduced abundance of SCFA-producing bacteria, particularly butyrate-producing taxa such as *Faecalibacterium*, *Roseburia*, and *Eubacterium* (Qin et al., 2012). Butyric acid is responsible, amongst other things, for stabilizing the intestinal barrier by promoting the expression of tight junction proteins such as claudin-3 and zonula occludens-1, and by increasing mucin production. Reduced butyric acid production, as observed in individuals with T2D or impaired glucose tolerance, can compromise intestinal barrier integrity, as demonstrated in studies using human intestinal epithelial cell model (Gaudier et al., 2004). SCFAs also contribute to the regulation of insulin sensitivity and the regulation of carbohydrate homeostasis partly by activating G protein-coupled receptors in the gut, including GPR41, GPR43, and GPR109a (Cunningham et al., 2021). Activation of these receptors enhances the release of GLP-1 and PYY, as shown in mouse models (Christiansen et al., 2018). GLP-1 stimulates insulin secretion, inhibits gastric emptying, increases hepatic glycogen storage, and reduces *de novo* glucose production in hepatocytes, primarily through insulin-mediated mechanisms. PYY primarily inhibits intestinal motility, gastric emptying, and pancreatic secretion, and decreases food intake. Additionally, SCFAs may reduce fat accumulation by lowering PPAR-γ receptor expression and modulating the immune system to decrease inflammation, as demonstrated in mouse studies (den Besten et al., 2015).

Significant attention is given to the role of SCFAs in this article. SCFAs reach physiologically relevant concentrations in the large intestine, where acetate, propionate, and butyrate are present at millimolar levels and directly influence epithelial metabolism and immune regulation. In contrast, SCFA concentrations in peripheral blood are substantially lower, typically remaining in the low micromolar range, particularly for butyrate and propionate, due to extensive intestinal and hepatic metabolism. SCFAs achieve concentrations sufficient to locally activate GPR41 and GPR43 receptors in the intestine and portal circulation. Reported EC50 values for SCFA-mediated activation of GPR41 and GPR43 generally lie within the high micromolar to low millimolar range (approximately 0.1–1 mM, depending on the receptor subtype, ligand, and assay system). However, systemic peripheral concentrations may often remain near or below reported EC50 (half maximal effective concentration) ranges for receptor activation in experimental systems, potentially limiting robust receptor engagement under physiological conditions. This discrepancy is a frequently overlooked limitation when extrapolating the mechanisms of SCFA receptor activation from cell culture studies to human systemic physiology (Sankarganesh et al., 2025; Martin-Gallausiaux et al., 2021).

### Bile acids

Dysbiosis of the gut microbiota disrupts bile acid metabolism, notably by promoting the conversion of primary to secondary bile acids via bacterial enzymes, primarily dehydroxylases (Ridlon et al., 2016). Beyond facilitating lipid digestion, bile acids function as signaling molecules that regulate metabolic processes by activating specific receptors, including FXR and TGR5. FXR activation modulates lipid metabolism, including reduced triglyceride levels, enhanced fatty acid β-oxidation, and improved glucose tolerance and insulin sensitivity. Alterations in gut microbiota composition can impair FXR-dependent signaling, thereby contributing to metabolic disorders associated with insulin resistance and T2D (Mori et al., 2022). Experimental studies indicate that secondary bile acids also serve as ligands for the TGR5 receptor, whose activation supports intestinal barrier integrity and stimulates secretion of GLP-1, thereby enhancing insulin release (Gao et al., 2022). In dysbiotic conditions, altered secondary bile acid profiles may reduce TGR5 activation, thereby exacerbating insulin resistance and hyperglycemia (Gurung et al., 2020).

### Amino acids

The gut microbiota contributes to amino acids metabolism, particularly branched-chain amino acids (BCAA) and aromatic amino acids (AAA) metabolism. Human studies indicate that elevated concentrations of BCAA and AAA may predict the future development of T2D (Najafi et al., 2023). Increased levels of BCAA, including leucine, isoleucine and valine, have been observed in patients with T2D (Mosley et al., 2024). These amino acids can activate the mammalian target of rapamycin (mTOR)/p70 S6 kinase (p70S6K) pathway, which inhibits phosphorylation of the insulin receptor substrate and impairs insulin signalling, as demonstrated in skeletal muscle cell lines (Tremblay and Marette, 2001). Elevated blood BCAA levels may inhibit AMPK activation, leading to increased hepatic gluconeogenesis and decreased glucose uptake by peripheral tissues, ultimately increasing blood glucose levels, as shown in rat models (Saha et al., 2010). In cases of gut dysbiosis, altered activity of BCAA-degrading enzymes may lead to excessive BCAA accumulation in the bloodstream. Mouse model studies have identified certain bacterial strains, such as *Prevotella copri* and *Bacteroides vulgatus*, as contributors to increased BCAA biosynthesis (Gojda and Cahova, 2021). In individuals with insulin resistance or T2D, elevated BCAA concentrations may also result from impaired metabolism. Rodent studies have shown that increased plasma BCAA concentrations are associated with reduced activity and dysregulation of enzymes involved in BCAA catabolism, such as branched-chain amino acid transaminase and the branched-chain α-ketoacid dehydrogenase complex (She et al., 2007). Deficiencies in these enzymes impair lipid and glucose oxidation by contributing to mitochondrial dysfunction (Lerin et al., 2016). Similarly, concentrations of AAA, including tyrosine, tryptophan and phenylalanine, are often elevated in patients with T2D (Li et al., 2023). Indole propionate (IPA), a specific metabolite of tryptophan produced primarily by the gut microbiota, is closely linked to dietary fibre intake. Higher blood concentrations of IPA are associated with a lower risk of developing T2D and improved insulin secretion in humans (Tuomainen et al., 2018).

Elevated BCAA levels are consistently observed in insulin-resistant individuals and are strongly associated with metabolic dysfunction. However, current evidence does not support a direct causal relationship whereby physiological concentrations of BCAA alone are sufficient to induce insulin resistance in humans. Instead, elevated circulating BCAA are best interpreted as biomarkers of impaired amino acid metabolism, while potentially contributing to the modulation and progression of metabolic disease in a context-dependent manner (Neinast et al., 2019).

Although several mechanistic pathways linking microbiota-derived metabolites to insulin resistance have been identified, much of the supporting evidence comes from animal models and cell culture systems, with direct confirmation in humans limited.

## Gut microbiota and CVD

Cardiovascular diseases cover a wide spectrum of conditions, including hypertension, atherosclerosis, peripheral vascular disease, heart valve disease, and heart failure, among others. Recent studies on the relationship between gut microbiota and the development of CVD have led to significant advances in understanding the mechanisms of this relationship (Muttiah and Hanafiah, 2025). Undoubtedly, some of the major risk factors for the development of CVD are lipid disorders, insulin resistance, diabetes, obesity, and chronic low-grade inflammation (Haybar et al., 2019). The role of microbiota in the development of these disorders has been described above. This part will focus on the changes in gut microbiota and its metabolites in relation to the development and progression of atherosclerosis, hypertension, and heart failure.

### Atherosclerosis

Multiple factors contribute to the development of atherosclerosis, notably chronic inflammation driven by lipopolysaccharide (LPS) derived from a dysbiotic gut microbiota. The presence of LPS in the bloodstream promotes inflammation and foam cell formation by activating the Toll-like receptor 4 (TLR4)/myeloid differentiation factor 88 (MyD88) pathway. Additionally, bacterial DNA of possible intestinal origin has been detected in human atherosclerotic plaques (Nicolaou et al., 2012). Comparative analyses of the microbiota in patients with atherosclerosis have revealed significant differences from healthy individuals. Specifically, individuals with atherosclerosis exhibit increased abundance of *Streptococcus* and *Enterobacteriaceae* species (Zhu et al., 2023). In patients with coronary artery disease (CAD), elevated levels of *Lactobacillales* and *Clostridium subcluster XIVa* colonies have been observed in the gut microbiota (Emoto et al., 2017). Trimethylamine N-oxide (TMAO) represents a principal link between gut microbiota and atherosclerosis. Gut bacteria metabolize choline, phosphatidylcholine, and carnitine from dietary sources into trimethylamine, which is subsequently oxidized to TMAO in the liver (Bennett et al., 2013). Elevated plasma TMAO levels are associated with an increased risk of coronary plaque rupture (Tan et al., 2019). Several mechanisms have been identified through which elevated TMAO levels may promote pro-atherosclerotic effects. TMAO can upregulate macrophage scavenger receptors including CD36, destabilizing cholesterol metabolism and leading to foam cell formation, as demonstrated in mouse studies (Wang et al., 2011). Inhibition of CYP7A1 by TMAO limits bile acid synthesis, thereby reducing cholesterol excretion and reabsorption, as shown in mouse models (Koeth et al., 2013). Destabilization of the vascular endothelium, induced by TMAO-mediated activation of NF-κB (nuclear factor kappa B) and the inflammasome, increases expression of endothelial inflammatory factors in mice (Ma et al., 2017). In addition to upregulating inflammatory factors, TMAO may disrupt intercellular junction regulation and alter endothelial permeability by activating High Mobility Group Box 1 (HMGB1). The resulting endothelial dysfunction contributes to the initiation and progression of atherosclerotic changes, as demonstrated in mouse endothelial cell lines (Singh et al., 2019). TMAO may also enhance platelet hyperreactivity, promoting platelet aggregation and intravascular clot formation. This effect is partly mediated by increased intracellular calcium release, as observed in mouse models (Zhu et al., 2016). In contrast, SCFAs, another group of gut microbiota metabolites, exert anti-atherosclerotic effects by inhibiting inflammation, lowering cholesterol levels, and reducing lipid deposition (Tonch-Cerbu et al., 2025). Secondary bile acids may also confer protection against atherosclerosis, primarily by regulating cholesterol excretion through bile salt hydrolase activity and the FXR/CYP7A1 pathway (Lau et al., 2017).

### Hypertension

Hypertension is among the most prevalent medical conditions globally. Research has established an association between the gut microbiota and blood pressure regulation. Although studies involving patients with hypertension remain limited, evidence indicates that these individuals exhibit reduced microbial diversity compared to those with normal blood pressure (Yang et al., 2015). In addition, individuals with elevated blood pressure display a decreased abundance of butyrate-producing bacteria, such as Butyricimonas and *Fusobacterium* (Kim et al., 2018). Positive correlations have also been identified between the abundance of *Ruminococcaceae*, *Streptococcus,* and *Turicibacter* and blood pressure. Moreover, a higher *Bacillota/Bacteroidota* ratio has been reported in individuals with hypertension (Yang et al., 2015). Recognition of gut dysbiosis as a potential contributor to hypertension has prompted the development of experimental gut microbiota transplantation. Transferring microbiota from hypertensive individuals to healthy mice increases blood pressure in the recipients (Li et al., 2017). Among gut microbiota metabolites, SCFAs are implicated in blood pressure regulation. Animal studies have demonstrated that SCFAs influence blood pressure through interactions with G protein-coupled receptors, including GPR41 and olfactory receptor 78 (Olfr78). Activation of GPR41 by SCFAs is generally associated with lower blood pressure, whereas stimulation of Olfr78 may increase it. Notably, the EC50 values of SCFAs, representing the concentrations required to achieve 50% of the maximum effect, differ substantially between Olfr78 and GPR41 (Pluznick, 2014). This difference partly accounts for the simultaneous action of SCFAs on both receptors despite their opposing effects on blood pressure. High salt intake is a recognized risk factor for hypertension. Sodium absorption primarily occurs in the intestine, where the gut microbiota likely plays a significant role. Rodent studies have shown that a high-sodium diet (HSD) alters the composition of the gut microbiota (Hamad et al., 2022). Specifically, diet-induced hypertensive rats exhibit increased populations of *Erwinia* and Corynobacteriaceae, along with a reduction in *Anaerostipes* (Bier et al., 2018). The HSD is also associated with decreased *Lactobacillus murinus* populations and increased induction of type 17 T helper cells, both of which are correlated with hypertension development in mouse models (Wilck et al., 2017).

### Heart failure

Heart failure, the most common end-stage manifestation of various cardiovascular diseases, has recently been associated with alterations in the gut microbiota. Intestinal ischaemia resulting from reduced cardiac output may cause intestinal wall oedema, compromise the intestinal barrier, promote bacterial translocation and inflammation, and alter microbiota composition, potentially accelerating heart failure progression (Sandek et al., 2007). In patients with chronic heart failure, increased proliferation of pathogenic microorganisms, such as *Shigella*, *Salmonella*, *Campylobacter,* and *Yersinia enterocolitica,* has been documented. A positive correlation exists between heart failure severity and elevated colony counts of *Campylobacter*, *Shigella* and *Candida* (Pasini et al., 2016). Elevated TMAO concentrations have been observed in patients with heart failure, with higher TMAO levels associated with increased mortality and a greater need for heart transplantation (Kanitsoraphan et al., 2018). In murine models of heart failure induced by aortic stenosis, supplementation with choline (a TMAO precursor) and TMAO itself led to increased circulating TMAO, greater myocardial fibrosis, and worsened cardiac function (Organ et al., 2016). Studies in rat models indicate that TMAO exacerbates myocardial fibrosis and ventricular remodelling, thereby contributing to heart failure development (Li X. et al., 2019). Although the precise mechanisms remain unclear, TMAO may influence the heart through pathways such as activation of the NLRP3 inflammasome and the TGF-β/SMAD3 axis, which can promote myocardial fibrosis, as shown in mouse studies (Li Z. et al., 2019). Individuals with heart failure also exhibit reduced abundance of butyrate-producing bacteria, particularly from the *Lachnospiraceae* and *Ruminococcaceae* families (Trøseid et al., 2020). Another short-chain fatty acid, propionate, may attenuate cardiac hypertrophy and fibrosis, primarily by modulating regulatory T cells, as demonstrated in mouse studies (Bartolomaeus et al., 2019). In congestive heart failure, both the quantity and composition of microbiota-derived bile acids are altered, with studies reporting decreased concentrations of primary bile acids and increased levels of certain secondary bile acids (Trøseid et al., 2020). Additionally, the microbiota metabolite indoxyl sulfate is linked to myocardial fibrosis and cardiac remodelling, as evidenced in rat models (Lekawanvijit et al., 2010).

This chapter has frequently discussed the potential role of TMAO in the development of CVD. Current evidence indicates that circulating TMAO levels in patients with CVD are associated with adverse metabolic and cardiovascular outcomes, and elevated plasma concentrations have been consistently reported in individuals with atherosclerosis, heart failure, and other cardiovascular conditions. Higher TMAO levels are correlated with an increased risk of major adverse cardiovascular events and mortality (Schiattarella et al., 2017). However, it remains uncertain whether the concentrations of TMAO observed in humans are independently sufficient to directly induce cardiovascular or metabolic dysfunction. Renal function, dietary patterns, gut microbiota composition, and overall cardiometabolic health significantly influence circulating TMAO levels, complicating the establishment of direct causality. Consequently, TMAO is generally regarded as both a biomarker and a potential modulator of CVD progression, rather than a definitively established primary causal factor (Canyelles et al., 2023).

Several mechanisms linking microbiota-derived metabolites to the development of CVD have been identified. However, most mechanistic evidence originates from animal models and *in vitro* studies. Therefore, these pathways should currently be considered biologically plausible rather than fully established cause-and-effect processes in humans. Additional human studies are required to confirm their clinical significance.

## Gut microbiome and CKD

The relationship between gut microbiota and CKD is bidirectional. Uremic toxins circulating in the blood of patients with CKD contribute to the development of gut dysbiosis. Conversely, altered gut microbiota produces metabolites that, upon entering the bloodstream, may accelerate CKD progression (Noce et al., 2022). In progressive CKD, both quantitative and qualitative changes in the microflora coincide with intestinal barrier dysfunction and increased intestinal wall permeability. Elevated blood urea concentrations promote the colonisation and proliferation of microorganisms that utilise urea as an energy source, as demonstrated in rat models (Zhou et al., 2024). This process leads to increased ammonia release into the intestinal lumen. Ammonia has been shown to increase intestinal permeability by altering intercellular junctions, as evidenced in studies on human colorectal cancer cell lines (Yokoo et al., 2021). Additional factors contributing to gut microbiota dysbiosis in CKD include increased antibiotic use, iron therapy for anaemia, increased delivery of undigested protein to the colon, and a tendency towards constipation (Cigarran Guldris et al., 2017). Regardless of the underlying cause, the gastrointestinal tract of patients with CKD exhibits proliferation of *Enterobacteriae*, *Enterococci*, *Lachnospiraceae* and *Ruminococcaceae*, alongside a decline in *Lactobacillaceae*, *Prevotellaceae*, *Bacteroidaceae* and *Bifidobacterium* spp. (Tourountzis et al., 2022). An increase in the production of intestinal urea metabolites is also observed (Beker et al., 2022). The combined effects of microbial metabolite production, compromised intestinal barrier, and neuroendocrine immune system, may contribute to CKD progression and the development of complications in the context of gut dysbiosis (Chi et al., 2021). Key microbial metabolites implicated include indoxyl sulfate (IS), p-cresol sulfate (pCS) and TMAO, which may play significant roles in CKD pathogenesis and complication development (Tsuji et al., 2024). Blood concentrations of these metabolites are elevated in patients with CKD, as demonstrated in multiple studies (Pelletier et al., 2019; Rodríguez-García et al., 2025). Indoxyl sulfate, produced via hepatic metabolism of indole (a tryptophan metabolite), has been linked to peripheral vascular disease and thrombosis in rat studies (Karbowska et al., 2018). Experimental studies in rodents indicate that IS may influence the expression of genes involved in renal interstitial fibrosis, specifically TGF-β1 and tissue inhibitor of metalloproteinase 1 (TIMP-1) (Miyazaki et al., 1997). Increased IS concentrations have also been observed in patients with CKD, correlating with worsening renal function (Wu et al., 2011). p-Cresol sulfate, converted in the liver from p-cresol (a product of tyrosine and phenylalanine fermentation), induces reactive oxygen species production in rat studies via NADPH oxidase activation and increased caspase-3 activity, thereby promoting apoptosis (Watanabe et al., 2013). A study using mouse proximal renal tubule cells has shown that IS and pCS activate the intrarenal RAAS and promote profibrotic processes leading to renal interstitial fibrosis and the progression of glomerular damage (Sun et al., 2012). Trimethylamine N-oxide, formed from choline, phosphatidylcholine, and L-carnitine, is negatively correlated with GFR in CKD patients (Jiang J. et al., 2025). Studies in mice demonstrate that TMAO increases SMAD3 phosphorylation (a key regulator of fibrosis) and may elevate the risk of coronary artery disease by exacerbating atherosclerosis and thrombosis (Wu et al., 2020). Additionally, mouse models indicate that rising TMAO levels are associated with worsening interstitial renal fibrosis (Sun et al., 2017). The gut microbiota also interacts with the nervous system through the production of various hormones and neurotransmitters (Pires et al., 2024). Experimental studies indicate that the families *Bifidobacteriaceae, Lactobacillaceae,* and *Prevotellaceae* are involved in synthesizing neurotransmitters such as GABA and acetylcholine, as well as promoting the production of incretins, including GLP-1, GLP-2, and PYY. GABA has been shown to stimulate natriuresis mainly due to inhibition of renal sympathetic nerve activity in rat kidney studies (Onal et al., 2019). Additionally, acetylcholine and GLP-1 may exert vasodilatory effects on blood vessels and reduce angiotensin II levels, thereby enhancing glomerular filtration, as observed in human studies (Skov et al., 2013; Wierema et al., 1997). In CKD, the abundance of these bacterial families is diminished, which has been proposed to contribute to activation of the RAAS, increased sympathetic tone, and the progression of hypertension and CKD (Sun et al., 2022).

This section discusses the protein-bound uremic toxins: IS and pCS. The concentrations of these microbial metabolites increase substantially in patients with advanced chronic kidney disease compared to healthy individuals, as impaired kidneys and dialysis are both ineffective at clearing them due to their strong binding to albumin. Elevated plasma levels of IS and pCS are associated with increased oxidative stress, inflammation, endothelial dysfunction, vascular calcification, and the progression of renal fibrosis. Experimental studies indicate that both toxins may contribute to renal and cardiovascular damage. However, most evidence of their pathogenic effects comes from *in vitro* experiments and animal models. Therefore, caution is necessary when extrapolating these findings to humans, as the direct relevance of these mechanisms to human pathophysiology remains incompletely established (Leong and Sirich, 2016; Gryp et al., 2017). For IS and pCS, no standardized or broadly comparable EC50 values describing their biological effects in pathophysiological models are available. The available experimental data describe cellular responses as a function of applied concentration, without reference to unified pharmacodynamic parameters. This limits the possibility of direct quantitative comparison with *in vivo* exposure and requires caution when interpreting concentration–effect relationships.

## Low-grade inflammation in metabolic disorders

Chronic, low-grade inflammation is strongly associated with various metabolic disorders, including obesity and T2D (Cani et al., 2007). Gut dysbiosis, defined by an overgrowth of Gram-negative bacteria, can exacerbate inflammation and accelerate disease progression. Gram-negative bacteria are the primary source of the pro-inflammatory factor, LPS, which must enter the systemic circulation to induce inflammation (Noor et al., 2023). Elevated circulating levels of LPS, often referred to as metabolic endotoxemia, have been reported in obesity and type 2 diabetes (Creely et al., 2007; Boutagy et al., 2016). Although findings in human studies are not entirely consistent and methodological differences complicate comparisons across studies, accumulating evidence suggests that metabolic endotoxemia may contribute to chronic low-grade inflammation associated with metabolic disorders. (Boutagy et al., 2016; Gomes et al., 2017). In experimental animal models, a chronic approximately two- to threefold increase in plasma LPS has been shown to induce low-grade systemic inflammation and hepatic insulin resistance, supporting a mechanistic link between metabolic endotoxemia and metabolic dysfunction (Cani et al., 2007). In mice, high-fat feeding impairs intestinal barrier function, facilitating the translocation of LPS into the circulation (Cani et al., 2008). Alterations in the gut microbiota, such as reduced *Akkermansia muciniphila* abundance in obese or high-fat diet-fed mice, are linked to decreased intestinal barrier integrity (Everard et al., 2013), which may facilitate LPS translocation into the systemic circulation. LPS can further indirectly disrupt barrier integrity by activating the toll-like receptor (TLR) 4-dependent pathway, which involves MyD88/IRAK 4 (interleukin-1 receptor-associated kinase 4) in enterocytes, promoting its movement into the bloodstream, as demonstrated in studies using human cell lines and mice (Guo et al., 2015). Research with human Caco-2 intestinal cell lines has shown that a high-fat diet increases LPS release into chylomicrons and enhances its passage from the gut into the circulation via the lymphatic system (Tomassen et al., 2023). Once in the systemic circulation, LPS binds to lipopolysaccharide-binding protein, forming a complex with CD14. This complex interacts with TLR4 receptors on macrophages or adipocytes, inducing the expression of activator protein 1 and NF-κB, which subsequently triggers the release of pro-inflammatory cytokines and chemokines, including tumour necrosis factor α (TNF-α), interleukin 6 (IL-6) and monocyte chemotactic protein 1 (Rogero and Calder, 2018). SCFAs, particularly butyrate, possess potent anti-inflammatory properties and counteract LPS effects by promoting regulatory T cells (Tregs) and differentiating IL-10-producing T cells via GPR109a. Butyrate-induced signalling through GPR109A also increases IL-18 secretion, a cytokine that supports intestinal epithelial integrity, as demonstrated in mouse studies (Liu et al., 2018). Additionally, studies using human Caco-2 intestinal cells and mouse colon cell cultures have shown that SCFAs inhibit TNF-α-induced NF-κB activation (Hung and Suzuki, 2018). However, it remains uncertain whether the anti-inflammatory properties of SCFAs are sufficient to attenuate LPS-induced chronic inflammation.

Chronic, low-grade inflammation significantly influences lipid metabolism. This phenomenon is partially attributed to LPS, which can enter the bloodstream due to gut dysbiosis and increased intestinal permeability. Exposure to LPS stimulates the production of pro-inflammatory cytokines, including TNF-α and IL-6, which contribute to insulin resistance by activating stress-related kinases that promote serine phosphorylation of insulin receptor substrate 1 (IRS-1) and thereby inhibit insulin signalling (Chen et al., 2015). Phosphorylation of rat IRS-1 at Ser307 disrupts its interaction with the insulin receptor, thereby impairing insulin signalling, as demonstrated using a yeast three-hybrid assay (Aguirre et al., 2002). Consequently, altered insulin signalling, is associated with adipose tissue dysfunction and hepatic lipid accumulation, which may further exacerbate insulin resistance and metabolic dysfunction (Petersen and Shulman, 2018). Furthermore, metabolic endotoxemia, characterized by elevated circulating LPS levels, may influence adipocyte hypertrophy and adipocyte precursor cell function through CD14- and activin A-dependent mechanisms, as demonstrated in mouse models (Luche et al., 2013; Gomes et al., 2018). Collectively, these processes may promote excessive lipid accumulation in adipose tissue and the liver, thereby contributing to the development and progression of obesity and its associated metabolic complications.

A compromised intestinal barrier in patients with chronic kidney disease can facilitate the translocation of gut microbiota-derived products, such as LPS, into the systemic circulation (Ramezani and Raj, 2014). TLR4 activation in macrophages caused by LPS can promote islet inflammation. Nackiewicz et al. (2014) showed that TLR4-activated macrophages contribute to IL-1α/IL-1β expression and IL-1β secretion in mouse and human islets, and that activated macrophages impair beta-cell insulin gene expression and insulin secretion partly through IL-1β- and IL-6-mediated mechanisms. Additionally, disturbances in the gut microbiota in individuals with chronic kidney disease may reduce the secretion of other metabolites, such as SCFAs (Wang et al., 2023). Reduced butyrate levels, a key SCFA, are associated with increased inflammation and further compromise of the intestinal barrier (Corte-Iglesias et al., 2024).

Recent research on the structural heterogeneity of lipopolysaccharides indicates that variations in LPS structure elicit distinct immune responses (Saha et al., 2022). Hexaacylated LPS, such as that from *E. coli*, is a strong TLR4 agonist that promotes glycaemic disturbances and adipose tissue inflammation, contributing to T2D (Rogero and Calder, 2018). In contrast, non-acylated LPS, such as from R. sphaeroides, acts as a TLR4 antagonist, does not impair glycaemic control at equal doses, and may even improve insulin sensitivity in obese mice (Anhê et al., 2021). Studies indicate that in healthy individuals, LPS with reduced acylation primarily induces TLR4 antagonism in the gut microbiota. This suggests that the overall metabolic effect of gut-derived LPS may be tolerogenic. In mice, oral LPS supplementation increased adiponectin production in adipose tissue and enhanced insulin signalling, potentially helping prevent T2D (Yamamoto et al., 2023). However, changes in the composition of the gut microbiota associated with obesity and T2D result in an increased abundance of Gram-negative bacteria, especially from the *Pseudomonadota* phylum (mainly from *Enterobacteriaceae* family), which generate highly immunostimulatory hexacylated lipopolysaccharides (Anhê et al., 2020).

Although LPS concentrations reported in metabolic endotoxemia are significantly lower than those observed in sepsis, they have been proposed to be sufficient to engage TLR4 signalling and contribute to chronic low-grade inflammation (Mohammad and Thiemermann, 2021). Chronic exposure to LPS has been associated with insulin resistance, non-alcoholic fatty liver disease, endothelial dysfunction, and the development of obesity and atherosclerosis although the causal contribution and physiological relevance of circulating gut-derived LPS remain debated (Mohammad and Thiemermann, 2021; Chen and Gautron, 2025). Furthermore, measuring LPS concentrations and assessing metabolic endotoxemia pose considerable methodological challenges. The Limulus Amebocyte Lysate (LAL) assay, which is most commonly employed, exhibits limited specificity and is highly susceptible to contamination; its results do not consistently reflect the actual biological activity of endotoxins. Additionally, a substantial proportion of circulating LPS is bound to lipoproteins, complicating accurate evaluation of its bioavailability and pro-inflammatory potential (Munford, 2016). Therefore, interpretation of LPS levels in patients with metabolic diseases should be approached with caution.

**FIGURE 1:**
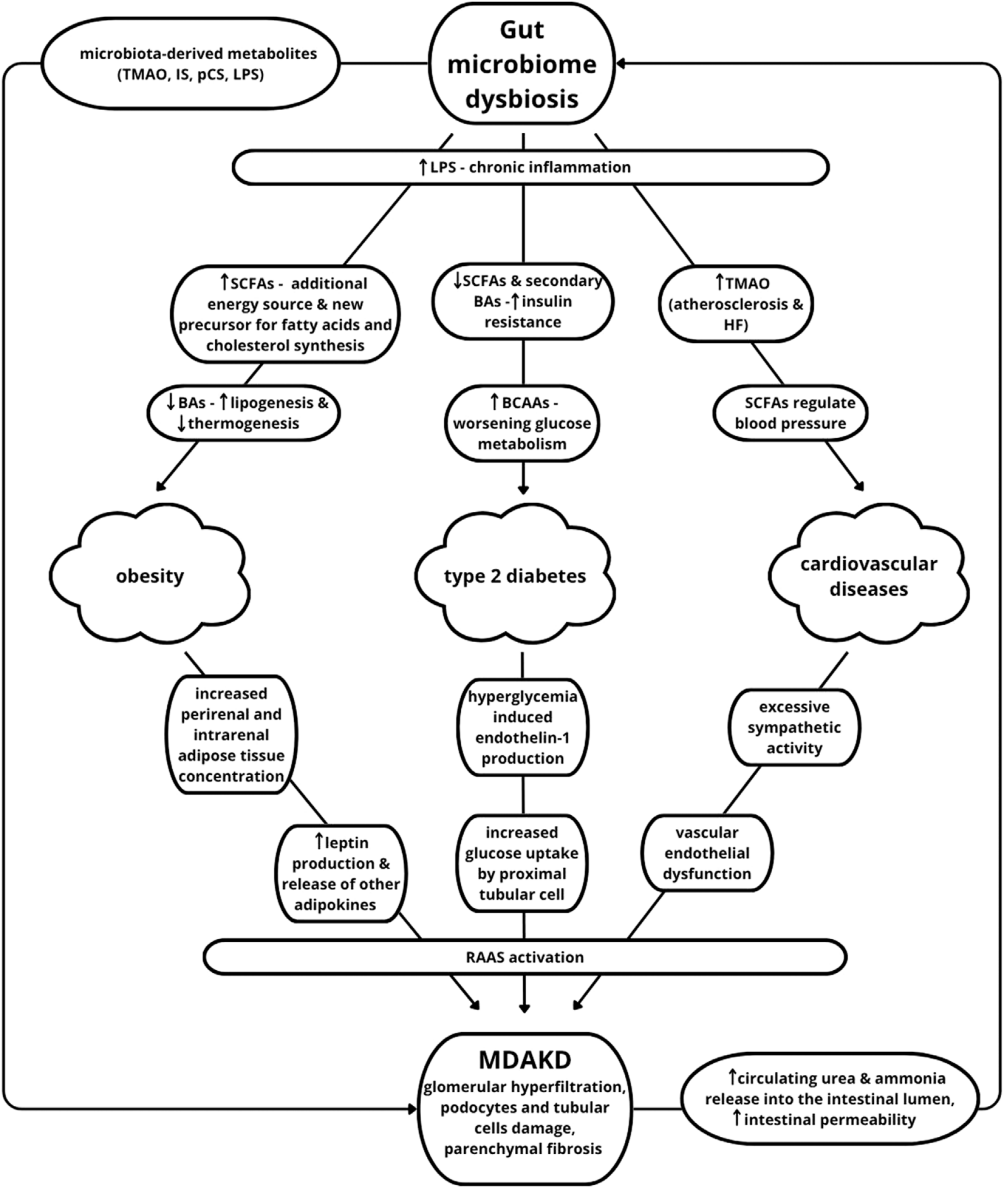
Gut microbiome dysbiosis as a potential trigger for metabolic dysfunction-associated kidney disease (MDAKD). The diagram depicts the potential association between gut microbiota dysbiosis and the onset of metabolic dysfunction-associated kidney disease (MDAKD), as outlined in the article. Alterations in gut microbiota composition may facilitate the release of lipopolysaccharides (LPS), thereby intensifying chronic inflammation. Variations in the concentrations of short-chain fatty acids (SCFAs), bile acids (BAs), and branched-chain amino acids (BCAAs) can disrupt lipid and glucose metabolism, leading to obesity, insulin resistance, and type 2 diabetes. Additionally, increased trimethylamine N-oxide (TMAO) levels and impaired blood pressure regulation by SCFAs may contribute to the development of cardiovascular disease and heart failure (HF). These metabolic disorders subsequently promote MDAKD through mechanisms such as endothelial dysfunction, adipose tissue remodeling, impaired renal tubular function, and activation of the renin-angiotensin-aldosterone system (RAAS). MDAKD is characterized by glomerular hyperfiltration, podocyte injury, and parenchymal fibrosis. Moreover, microbiota-derived metabolites, including indoxyl sulfate (IS) and p-cresyl sulfate (pCS), may contribute to kidney injury. Elevated urea and ammonia levels observed in chronic kidney disease (CKD) further increase intestinal permeability, thereby worsening adverse alterations in the gut microbiota. Abbreviations: MDAKD, metabolic dysfunction-associated kidney disease; LPS, lipopolysaccharide; SCFAs, short-chain fatty acids; BAs, bile acids; BCAAs, branched-chain amino acids; TMAO, trimethylamine N-oxide; IS, indoxyl sulfate; pCS, p-cresyl sulfate; HF, heart failure; RAAS, renin–angiotensin–aldosterone system; CKD, chronic kidney disease.

To summarise, Figure 1 presents a concise overview of the potential mechanisms previously described that connect disturbances in gut microbiota balance to the development of MDAKD.

## Prevention and potential treatmentapproach

Given the potential role of the gut microbiota in the development of MDAKD, this study aims to present a new perspective on the treatment and progression of CKD. Dysbiosis of the gut microbiota is linked to metabolic endotoxemia, characterized by elevated concentrations of LPS translocating from the gastrointestinal tract into systemic circulation, where LPS impairs insulin signaling in key tissues such as adipose and muscle. Achieving eubiosis in the gut microbiota can mitigate or prevent this process by enhancing tissue insulin sensitivity, improving glucose metabolism, and reducing low-grade inflammation (Cani et al., 2007). Addressing gut dysbiosis is therefore a potentially important component of obesity management. Restoration of gut microbiota balance stimulates the secretion of satiety hormones, including PYY and GLP-1, by L cells in the gut (Du et al., 2024). Intake of prebiotics, such as inulin, fructooligosaccharides, and resistant starch (found in unripe bananas and chilled potatoes), supports the growth of *Bifidobacteria* and *Lactobacilli, which may positively influence* kidney function (Mao et al., 2023). A systematic review has underscored the role of probiotics in reducing plasma concentrations of urea, blood urea nitrogen, ammonia, IS, and pCS in individuals with CKD, with strains from the genera *Lactobacillus* and Bifidobacterium being most effective. However, due to the limited number of studies, these findings cannot yet be generalized, and further long-term research is required to clarify the potential role of probiotics in CKD management (Fagundes et al., 2018). Probiotics containing *Akkermansia muciniphila* may enhance intestinal barrier integrity and alleviate symptoms of leaky gut syndrome, as demonstrated in cell culture studies (Shi et al., 2022). Current dietary guidelines recommend a daily intake of 25–35 g of dietary fiber, which facilitates the production of butyrate, a key SCFA, through anaerobic fermentation of complex carbohydrates (Prasad and Bondy, 2018). A meta-analysis evaluating interventions to reduce protein-bound uremic toxins in CKD patients found that regular administration of prebiotics, synbiotics, and AST-120 (an oral, spherical carbon adsorbent for intestinal use) significantly lowers serum IS and pCS concentrations compared to placebo (Takkavatakarn et al., 2021). This approach may also decrease renal scarring. Contemporary therapeutic strategies for obesity and diabetes include GLP-1 receptor agonists and sodium-glucose co-transporter 2 (SGLT-2) inhibitors. SGLT-2 inhibitors modulate systemic glucose metabolism by inducing glucosuria, thereby reducing glucose availability in the intestinal lumen, limiting its use during anaerobic saccharolytic fermentation, and altering microbiota composition. This includes restoring the Bacillota/Bacteroidota ratio and increasing the abundance of SCFA-producing Lachnospiraceae. Additionally, SGLT-2 inhibitors reduce bacteria that produce IS and pCS, as shown in a study of 90 patients with CKD (Hsu et al., 2025). GLP-1 analogs, such as liraglutide, may attenuate glomerular inflammation and fibrosis in DKD by modulating the TLR4 signaling pathway, as demonstrated in a rat glomerular mesangial cell line (Huang et al., 2024). In rat models of diabetic nephropathy, liraglutide exhibited nephroprotective effects by improving gut microbiota composition and increasing serum L-5-oxoproline concentrations. L-5-oxoproline was also found to significantly reduce ectopic lipid deposition in renal tubular cells by inhibiting lipid synthesis (Yi et al., 2024). In steatotic kidneys, excessive accumulation of triglycerides and cholesterol in podocytes and tubular cells induces lipotoxicity, as demonstrated in both animal models and humans with DKD. This process promotes oxidative stress and inflammation, ultimately resulting in renal cell death, fibrosis, and loss of filtration function (Schelling, 2022). A network meta-analysis has shown that probiotic supplementation may improve health outcomes and potentially slow the progression of CKD, although the evidence for anti-inflammatory and other pleiotropic effects remains inconclusive. The analysis further suggested that multi-strain formulations containing three or more probiotic strains may be associated with greater improvements in kidney function, metabolic parameters, inflammation, and oxidative stress in patients with CKD (Li et al., 2025). However, this finding should be interpreted with caution, as probiotic efficacy is considered highly strain- and disease-specific, and the included interventions were grouped primarily by the number of strains rather than their individual identities (McFarland et al., 2018). Therefore, further well-designed randomized controlled trials are needed to determine which specific probiotic strains are effective for particular CKD-related outcomes and to establish evidence-based recommendations for clinical practice (Liu et al., 2024). Fecal microbiota transplantation (FMT) is a distinct therapeutic approach, separate from probiotics or dietary interventions. It has primarily been used for recurrent *Clostridioides difficile* infections, with an effectiveness exceeding 80%. Emerging research indicates that FMT may also benefit patients with CKD. In mouse studies, FMT from healthy control mice treated with resveratrol restored the gut microbiota, regulated intestinal permeability, reduced inflammation, and improved kidney function (Cai et al., 2020). Another study used rat models, performing FMT from healthy rats to those with streptozotocin-induced diabetes. Microbiota transplantation significantly reduced tubular injury in diabetic rats. This improvement was linked to restored cholesterol homeostasis, lower serum triglyceride levels, and decreased lipid accumulation in the kidneys, mediated by GPR43 activation (Hu et al., 2020). FMT has also been applied in humans to improve kidney function. In a clinical case report, a patient with membranous nephropathy received an endoscopic transplant of gut microbiota from a healthy donor. After two FMT procedures, the patient’s nephrotic syndrome was alleviated, and kidney function improved, as indicated by increased serum total protein and albumin, and decreased serum creatinine and 24-h urine protein levels (Zhou et al., 2021). A comprehensive approach involving a suitable diet, probiotics, and pharmacological treatment may help protect against metabolic toxicity and kidney deterioration. However, further research and standardization are needed before FMT can be recommended for chronic kidney disease.

## Conclusion

The gut microbiota is increasingly recognised as a potentially important contributor to the pathogenesis of kidney diseases associated with metabolic disorders, due to its influence on metabolic, inflammatory, immunological, and neuroendocrine pathways. Microbial dysbiosis may contribute to the development and progression of obesity, type 2 diabetes, cardiovascular diseases, and chronic kidney disease by altering the production of microbial metabolites, compromising intestinal barrier integrity, and promoting a chronic, low-grade inflammatory state. Key metabolites derived from the microbiota, such as short-chain fatty acids, bile acids, trimethylamine N-oxide, indoxyl sulfate, p-cresol sulfate, branched-chain amino acids, and lipopolysaccharides, have been shown to modulate metabolic and renal functions through diverse mechanisms. Although substantial progress has been made in elucidating the gut-kidney axis, several critical uncertainties persist. Many of the mechanistic pathways discussed in this review should be regarded as biologically plausible hypotheses supported by preclinical evidence rather than fully established causal mechanisms in humans. Additionally, pronounced inter-individual variability in microbiota composition, methodological challenges in metabolite quantification, and the complexity of host-microbiota interactions complicate the interpretation of current findings. Importantly, receptor-specific EC50 values are only well established for selected gut-derived metabolites such as SCFAs, whereas for most other microbiota-derived compounds discussed in this review, pharmacodynamic thresholds remain undefined or assay-dependent. Therefore, many microbiota-associated metabolites should presently be considered potential contributors to disease progression or biomarkers, rather than definitively established causative agents. Despite these limitations, accumulating evidence indicates that modulation of the gut microbiota may offer a promising therapeutic approach for metabolic and renal diseases. Dietary interventions, prebiotics, probiotics, pharmacological agents, and faecal microbiota transplantation have demonstrated potential benefits in experimental models and early-phase clinical trials, although their long-term efficacy and clinical applicability remain to be established. Rigorous, well-designed human studies are required to clarify underlying mechanisms, identify clinically relevant microbiological targets, and determine whether microbiota-targeted therapies can effectively prevent or slow the progression of MDAKD.

## Glossary

AAA: aromatic amino acids
AGRP: Agouti-related protein
AMPK: AMP-activated kinase
AST-120: oral, spherical carbon adsorbent for intestinal use
BCAA: branched-chain amino acids
CD36/CD14: cluster of differentiation 36/14
ChREBP: carbohydrate response element binding protein
CKD: chronic kidney disease
CVD: cardiovascular disease
DKD: diabetic kidney disease
DNA: deoxyribonucleic acid
EC50: half maximal effective concentration
ESRD: end-stage renal disease
FGF19: fibroblast growth factor 19
FIAF/ANGPTL4: fasting-induced adipose factor
FMT: fecal microbiota transplantation
FXR: farnesoid X receptor
GABA: γ-aminobutyric acid
GFR: glomerular filtration rate
GLP-1/2: glucagon-like peptide 1/2
GLUT2: glucose transporter 2
GPR41/43/109a: G protein coupled receptor 41/43/109a
HSD: high-sodium diet
IL-1β/6/10/18: interleukin 1β/6/10/18
IPA: indolepropionate
IRAK4: interleukin-1 receptor-associated kinase 4
IS: indoxyl sulfate
LAL: Limulus Amebocyte Lysate
LPS: lipopolysaccharide
MDAKD: metabolic dysfunction-associated kidney disease
MyD88: myeloid differentiation factor 88
NADPH: nicotinamide adenine dinucleotide phosphate, reduced form
NF-κB: nuclear factor kappa B
Olfr78: olfactory receptor 78
pCS: p-cresol sulfate
PPAR-γ: proliferator–activated receptor γ
PYY: peptide YY
RAAS: renin–angiotensin–aldosterone system
SCFA/SCFAs: short-chain fatty acid/acids
SGLT-2: sodium-glucose linked transporter 2
SMAD3: mothers against decapentaplegic homolog 3
SREBP1c: sterol regulatory binding protein1c
T2D: type 2 diabetes
TGF-β: transforming growth factor beta
TGR5: takeda G protein-coupled receptor 5
TIMP-1: tissue inhibitor of metalloproteinase 1
TLR4: toll-like receptor 4
TMAO: trimethylamine *N*-oxide
TNF-α: tumor necrosis factor α
UCP1: uncoupled protein 1
